# Complete genome sequence and analysis of a novel lymphocystivirus detected in whitemouth croaker (*Micropogonias furnieri*): lymphocystis disease virus 4

**DOI:** 10.1007/s00705-020-04570-1

**Published:** 2020-03-05

**Authors:** Andor Doszpoly, Győző L. Kaján, Rodrigo Puentes, Alejandro Perretta

**Affiliations:** 1 Centre for Agricultural Research, Institute for Veterinary Medical Research, Hungarian Academy of Sciences, P.O. Box 18, Budapest, 1581 Hungary; 2 Instituto de Patobiología, Facultad de Veterinaria, Universidad de la República, Montevideo, Uruguay; 3 Instituto de Investigaciones Pesqueras, Facultad de Veterinaria, Universidad de la República, Montevideo, Uruguay

## Abstract

A novel lymphocystivirus causing typical signs of lymphocystis virus disease in whitemouth croaker (*Micropogonias furnieri*) on the coast of Uruguay was detected and described recently. Based on genetic analysis of some partially sequenced core genes, the virus seemed to differ from previously described members of the genus *Lymphocystivirus*. In this study, using next-generation sequencing, the whole genome of this virus was sequenced and analysed. The complete genome was found to be 211,086 bp in size, containing 148 predicted protein-coding regions, including the 26 core genes that seem to have a homologue in every iridovirus genome sequenced to date. Considering the current species demarcation criteria for the family *Iridoviridae* (genome organization, G+C content, amino acid sequence similarity, and phylogenetic relatedness of the core genes), the establishment of a novel species (“*Lymphocystis disease virus 4*”) in the genus *Lymphocystivirus* is suggested.

**Electronic supplementary material:**

The online version of this article (10.1007/s00705-020-04570-1) contains supplementary material, which is available to authorized users.

Lymphocystis disease viruses (LCDVs) belong to the genus *Lymphocystivirus*, family *Iridoviridae*. LCDVs are large (200-230 nm) dsDNA viruses. Their genome, which ranges in size between 108 and 208 kilobasepairs (kbp), is circularly permuted, terminally redundant, and heavily methylated at cytosines in CpG sequences, and these viruses typically have a low G+C content (27-33%) [4, 7]. LCDVs infect a wide range of fish species (over 150). A common disease sign is the development of macroscopic nodules (0.3-2.0 mm) located on the body surface and fins [1]. The genus *Lymphocystivirus* currently includes three virus species accepted by the International Committee on Taxonomy of Viruses (ICTV): *Lymphocystis disease virus 1*, *2* and *3*. The complete genome sequence of the LCDV-1 was published in the 1990s [14], and this virus has been reported in flatfishes (family Pleuronectidae) [3]. LCDV-2 was isolated also from a flatfish species, namely flounder (*Paralichthys olivaceus*) [15], while LCDV-3 was found in gilthead sea bream (*Sparus aurata*) [11].

A few years ago, an outbreak of lymphocystis disease (LCD) was detected in wild and cultured populations of whitemouth croaker (*Micropogonias furnieri*) on the coast of Uruguay. Molecular analysis targeting some of the iridoviral core genes showed the presence of the DNA of an unknown LCDV in all specimens showing external signs of LCD. Phylogenetic analysis based on the concatenated sequences of six partially sequenced core genes suggested that the virus belongs to the genus *Lymphocystivirus*. However, the sequences of the whitemouth croaker LCDV (LCDV-WC) differed markedly from those of members of the three accepted species in this genus, putatively representing a fourth viral species in the genus. In the present study, using next-generation sequencing, the whole genome of this virus was sequenced and analysed.

Diseased fish were collected on the coast of Uruguay [13]. Samples from the lesions were conserved in ethanol for molecular studies. Total DNA was extracted from the samples using a DNeasy Blood and Tissue Kit (QIAGEN, Germany). A short-insert DNA library was prepared and sequenced using the Illumina HiSeqTM 2000 platform (Illumina, USA). A total of 16,834,698 paired-end reads were generated (Q20% = 96.33), with the average length of 66 bp; CLC Genomics Workbench 12.0 (CLC bio, Denmark) and Geneious 11.1.5 (Biomatters Ltd., New Zealand) were used for genome assembly (average read depth = 2916). There were two short gaps between the contigs. These gaps were closed and some further sequence ambiguities were resolved using Sanger sequencing with PCR primers designed based on the flanking regions of sequences. FGENESV was used for prediction of open reading frames (ORFs) (Softberry, Inc., USA). The complete genome sequence of the novel LCDV was deposited in the GenBank database under the accession number MN803438. The genome sequence was compared to those of LCDV-1, -2 and -3 using LASTZ 1.02.00 in Geneious 11.1.5 (Biomatters Ltd., New Zealand). In LASTZ, default settings were used: the high-scoring segment pairs—homologous stretches—were scored using the HOXD70 substitution scores [2], and the lower score threshold was 3000. The deduced amino acid sequences of the proteins encoded by the 26 core genes (Supplementary Table 1) were concatenated, and this sequence was used for phylogenetic analysis. For tree inference, a multiple alignment of the concatenates was made using Mafft v7 [10] with default parameters, and the alignment was edited manually. Evolutionary model selection was done using ModelTest-NG v0.1.5 [5], and the LG+I+G model had the highest probability. The phylogenetic calculation was performed using RAxML-NG v0.9.0 [8], the robustness of the tree was analyzed using a non-parametric bootstrap calculation with 1,000 repeats. The phylogenetic tree was visualized using MEGA 7 [9], and bootstrap values are given as percentages. A pairwise sequence identity analysis was also conducted on the same concatenate of the four LCDVs, using SDT 1.2 [12].

The complete genome of the LCDV-WC was found to be 211,086 bp in size. The G+C content of the whole genome was 26.0%. Comparison of the genome sequence of LCDV-WC to those of previously described LCDVs showed that the genome size of LCDV-WC is the longest, and its G+C content is the lowest (LCDV-1, 29.1%; LCDV-2, 27.2%; LCDV-3, 33.0%). The genome organization of the LCDV-WC shows similarity to that of LCDV-2 and -3, but major rearrangements are also observable (Fig. 1). The LCDV-WC genome was predicted to contain 148 ORFs. The majority of the ORFs (102) showed clear homology to the genes of all three other LCDVs. The 26 core genes, which are conserved in all sequenced iridoviruses, were also identified in the genome. Nine ORFs lacked similarity to any known viral gene. The rest of the putative genes showed homology to genes of only one or two of the LCDVs. The protein product concatenate of the core genes showed 67.1–85.1% amino acid sequence identity to its LCDV counterparts (Fig. 2). The phylogenetic tree reconstruction clearly illustrates that LCDV-WC clusters with members of the genus *Lymphocystivirus* and shows a clear separation of LCDV-WC from the other LCDVs (Fig. 3).

**Fig. 1:**
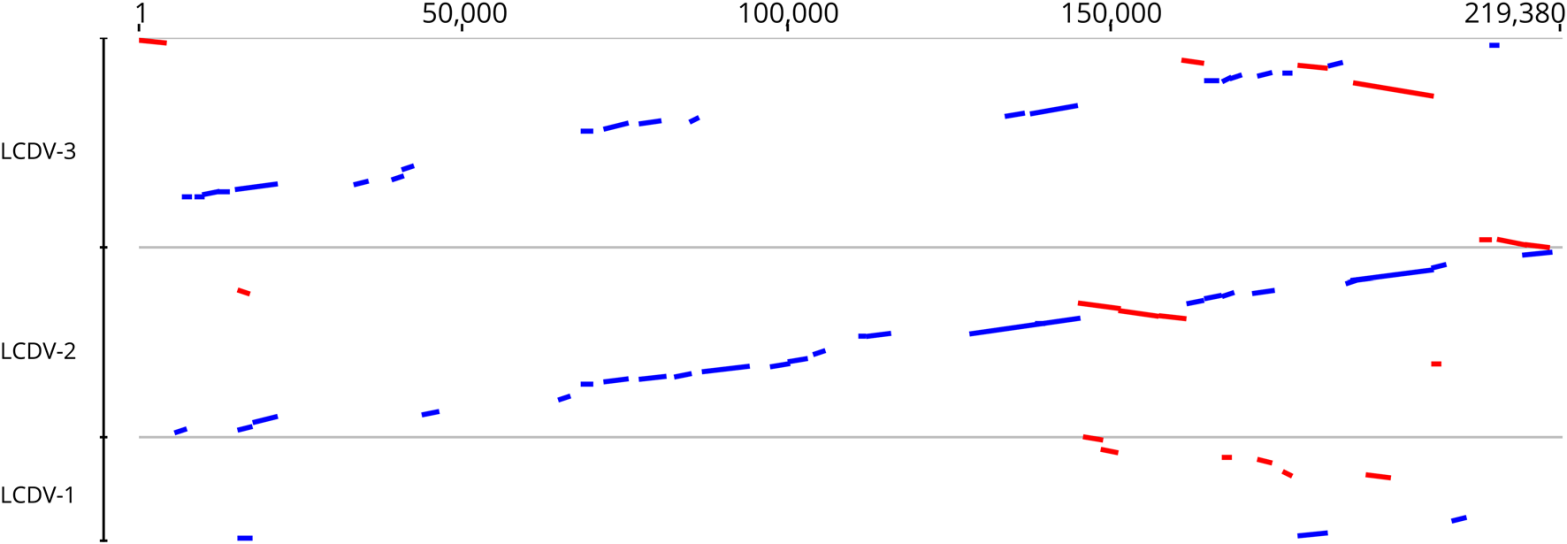
Comparison of the whitemouth croaker lymphocystis disease virus (LCDV-WC) genome sequence to those of *LCDV1-3*. The genome sequence of LCDV-WC is on the abscissa, and the genome sequences of LCDV1, LCDV2, and LCDV3 are on the ordinate in separate sections. Blue bars represent homologous high-scoring segment pairs in a codirectional orientation, whereas red bars represent reversed pairs. LCDV, lymphocystis disease virus

**Fig. 2:**
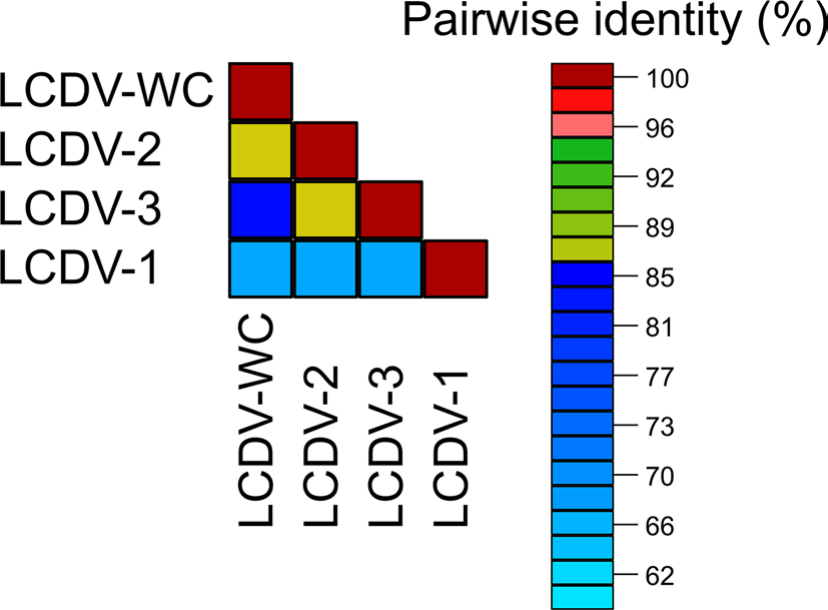
Pairwise sequence identity analysis of 26 core gene amino acid sequence concatenates of LCDV1-3 and whitemouth croaker lymphocystis disease virus. LCDV, lymphocystis disease virus; LCDV-WC, whitemouth croaker lymphocystis disease virus. Pairwise sequence identity percentages are represented using different colours. Cutoff values: 85% and 95%. Blue squares represent similarity below 85%; green squares, 85-95%; red squares, ≥95%

**Fig. 3:**
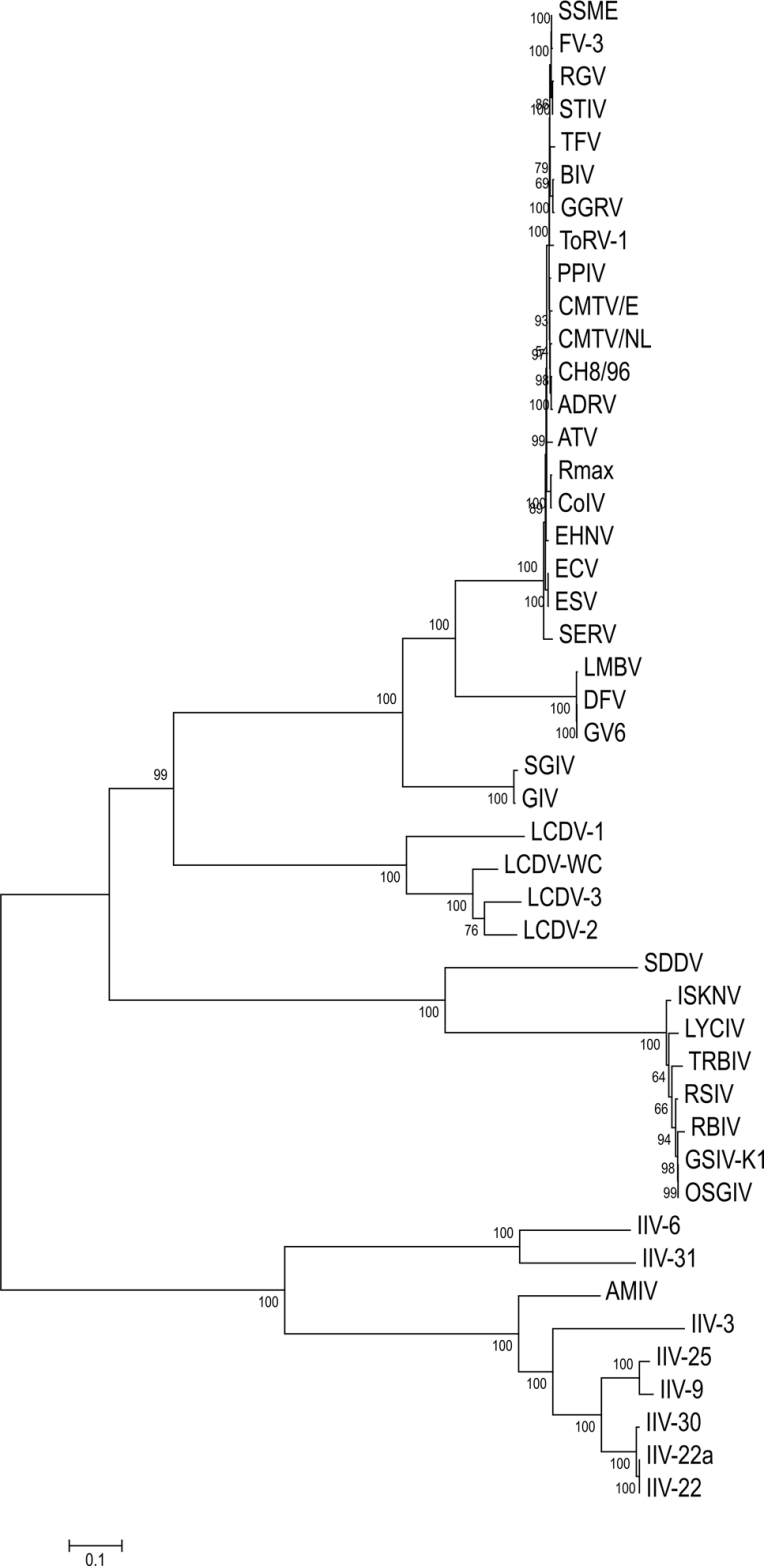
Phylogenetic analysis of iridoviruses. Maximum-likelihood analysis of 26 core gene amino acid sequence concatenates (8178 aa). The tree is rooted at the midpoint. Bootstrap values are given as percentages. ADRV, Andrias davidianus ranavirus; AMIV, Anopheles minimus iridovirus; ATV, Ambystoma tigrinum virus; BIV, Bohle iridovirus; CH, Testudo hermanni ranavirus; CMTV, common midwife toad virus; CoIV, cod iridovirus; DFV, doctor fish virus; ECV, European catfish virus; EHNV, epizootic haematopoietic necrosis virus; ESV, European sheatfish virus; FV, frog virus; GGRV, German gecko ranavirus; GIV, grouper iridovirus; GSIV, giant seaperch iridovirus; GV, guppy virus; IIV, invertebrate iridescent virus; ISKNV, infectious skin and kidney necrosis virus; LCDV, lymphocystis disease virus; LMBV, largemouth bass virus; LYCIV, lemon yellow croaker iridovirus; OSGIV, orange-spotted grouper iridovirus; PPIV, pike perch iridovirus; RBIV, rock bream iridovirus; RGV, Rana grylio virus; Rmax, Rana maxima virus; RSIV, red seabream iridovirus; SDDV, scale drop disease virus; SERV, short-finned eel ranavirus; SGIV, Singapore grouper iridovirus; SSME, spotted salamander Maine virus; STIV, soft-shelled turtle iridovirus; TFV, tiger frog virus; ToRV, tortoise ranavirus; TRBIV, turbot reddish body iridovirus

There are 26 well-conserved core genes in the genomes of all known and completely sequenced iridoviruses, the products of which are associated with a variety of viral activities, including DNA metabolism, transcriptional regulation, protein modification, and viral structure [6]. According to the current species demarcation criteria for the members of the family *Iridoviridae* (https://talk.ictvonline.org/files/ictv_official_taxonomy_updates_since_the_8th_report/m/animal-dna-viruses-and-retroviruses/8054), viruses sharing 95% or greater amino acid sequence identity in the predicted products of their core genes should be considered members of the same species. Moreover, members of the same species have to have a similar genome size and G+C content, and they should show phylogenetic relatedness and a collinear gene arrangement. The analysis of the complete genome sequence of LCDV-WC confirmed that this virus is a member of a distinct species in the genus *Lymphocystivirus*, as was suspected from partial sequence information from a previous study [13]. This demonstrates that complete genome sequences may not be necessary for establishing a novel species. The authors propose that the establishment of the new species “*Lymphocystis disease virus 4*” should be considered for approval by the ICTV.

## Electronic supplementary material

Below is the link to the electronic supplementary material.

Supplementary Table 1 Name, GenBank accession number, and locus tag of the 26 core genes in the genomes of lymphocystiviruses (DOCX 16 kb)
