# Supplementary material for: Complete genome sequence and analysis of a novel lymphocystivirus detected in whitemouth croaker (Micropogonias furnieri): lymphocystis disease virus 4
Source: Arch Virol. 2020 Mar 5;165(5):1215–8. doi: 10.1007/s00705-020-04570-1 (PMC7160068; doi:10.1007/s00705-020-04570-1)
Supplement: Supplementary file 1 — Supplementary Table 1 Name, GenBank accession number, and locus tag of the 26 core genes in the genomes of lymphocystiviruses (DOCX 16 kb) [file 705_2020_4570_MOESM1_ESM.docx]

|  | Gene Name | LCDV-1  Accession No.:  NC_001824.1 | LCDV-2  Accession No.:  NC_005902.1 | LCDV-3  Accession No.:  NC_033423.1 | **LCDV-4**  **Accession No.: MN803438** |
| --- | --- | --- | --- | --- | --- |
| 1. | Myristylated membrane protein | LCDV1gp089 | LDVICp038 | LCDVSa074R | **LCDV-WC-orf124** |
| 2. | DNA-dependent RNA polymerase largest subunit | LCDV1gp007 | LDVICp190 | LCDVSa161R | **LCDV-WC-orf022** |
| 3. | SWI/SNF2 family helicase protein | LCDV1gp070 | LDVICp075 | LCDVSa112L | **LCDV-WC-orf100** |
| 4. | Putative DNA repair protein RAD2 | LCDV1gp107 | LDVICp168 | LCDVSa167L | **LCDV-WC-orf041** |
| 5. | Hypothetical protein | LCDV1gp009 | LDVICp152 | LCDVSa090R | **LCDV-WC-orf050** |
| 6. | Hypothetical immediate-early protein | LCDV1gp024 | LDVICp161 | LCDVSa128R | **LCDV-WC-orf038** |
| 7. | Major capsid protein | LCDV1gp080 | LDVICp043 | LCDVSa062R | **LCDV-WC-orf128** |
| 8. | Ervl/Alr family protein | LCDV1gp055 | LDVICp141 | LCDVSa107L | **LCDV-WC-orf071** |
| 9. | Deoxynucleoside kinase | LCDV1gp073 | LDVICp027 | LCDVSa049R | **LCDV-WC-orf113** |
| 10. | Proliferating cell nuclear antigen | LCDV1gp002 | LDVICp196 | LCDVSa144L | **LCDV-WC-orf019** |
| 11. | Transcription elongation factor S II | LCDV1gp097 | LDVICp115 | LCDVSa069L | **LCDV-WC-orf082** |
| 12. | Ribonuclease III | LCDV1gp074 | LDVICp186 | LCDVSa153L | **LCDV-WC-orf024** |
| 13. | Ribonucleotide reductase | LCDV1gp015 | LDVICp041 | LCDVSa064L | **LCDV-WC-orf127** |
| 14. | DNA-dependent RNA polymerase II second largest subunit | LCDV1gp013 | LDVICp025 | LCDVSa060R | **LCDV-WC-orf138** |
| 15. | DNA polymerase elongation subunit family B | LCDV1gp072 | LDVICp202 | LCDVSa142R | **LCDV-WC-orf017** |
| 16. | Putative phosphotransferase | LCDV1gp077 | LDVICp177 | LCDVSa145L | **LCDV-WC-orf034** |
| 17. | Myristylated membrane protein | LCDV1gp035 | LDVICp157 | LCDVSa123R | **LCDV-WC-orf047** |
| 18. | Putative tyrosine kinase | LCDV1gp110 | LDVICp172 | LCDVSa165L | **LCDV-WC-orf043** |
| 19. | Putative NIF/NLI interacting factor | LCDV1gp043 | LDVICp147 | LCDVSa092L | **LCDV-WC-orf058** |
| 20. | Hypothetical protein | LCDV1gp092 | LDVICp234 | LCDVSa005R | **LCDV-WC-orf004** |
| 21. | Putative D5 family NTPase/ATPase | LCDV1gp069 | LDVICp078 | LCDVSa113L | **LCDV-WC-orf098** |
| 22. | Hypothetical protein | LCDV1gp004 | LDVICp007 | LCDVSa054R | **LCDV-WC-orf143** |
| 23. | Serine/threonine protein kinase | LCDV1gp005 | LDVICp013 | LCDVSa059L | **LCDV-WC-orf139** |
| 24. | Virion assembly protein, NTPase | LCDV1gp030 | LDVICp114 | LCDVSa068L | **LCDV-WC-orf080** |
| 25. | Hypothetical protein | LCDV1gp057 | LDVICp100 | LCDVSa067L | **LCDV-WC-orf077** |
| 26. | Putative replicating factor | LCDV1gp091 | LDVICp180 | LCDVSa147R | **LCDV-WC-orf031** |

**Supplementary Table 1**
